# The Efficacy of Bereavement Interventions: A Systematic Umbrella Review

**DOI:** 10.1097/HRP.0000000000000426

**Published:** 2025-03-31

**Authors:** Kate A. Avis, Marjolein Missler, Denise van Deursen, Lonneke I.M. Lenferink, Margaret Stroebe, Henk Schut

**Affiliations:** **From** Department of Clinical Psychology, Utrecht University (Ms. Avis and Drs. Missler, Stroebe, and Schut); Department of Clinical Psychology, Open University (Dr. van Deursen); Department of Psychology, Health & Technology, University Twente (Dr. Lenferink); Department of Clinical Psychology and Experimental Psychopathology, University of Groningen (Drs. Lenferink and Stroebe).

**Keywords:** bereavement, bereavement intervention, grief, narrative synthesis, umbrella review

## Abstract

**Learning Objectives:**

After participating in this CME activity, the psychiatrist should be better able to:

• Summarize findings from systematic reviews and meta-analyses on the efficacy of psychotherapeutic bereavement interventions.

• Identify and apply key moderating factors (e.g., symptom severity, timing, age, gender) that influence intervention outcomes.

• Analyze methodological limitations in the bereavement literature, including study design and review quality issues.

**Abstract:**

In recent decades, there have been diverse reviews published on intervention program value for bereaved people. The variation and multiplicity of such reviews makes it difficult to obtain an overview of what is known about treatment effectiveness. In this systematic umbrella review, we explore the current knowledge base on psychotherapeutic bereavement intervention program efficacy. Thirty-three quantitative systematic reviews and/or meta-analyses published between January 2001 and October 2021 were included. Quality was assessed using the Assessment of Multiple Systematic Reviews criteria. Intervention efficacy was determined by rating overall conclusions into three categories according to strength of evidence: positive-unconditional, positive-conditional, and negative-no evidence. Our results indicate that bereavement interventions are generally helpful. Seven reviews indicated positive-unconditional support for bereavement interventions. Twenty-four reviews found positive-conditional support (i.e., some evidence of value, but efficacy did not apply in all circumstances or was constrained by database weaknesses or weak effects), and only two reviews indicated negative-no evidence for support. Notably, conclusions were generally limited by poor review quality and methodological concerns (e.g., lack of randomized controlled trials and follow-up studies). As such, we call for future empirical studies and review articles to abide by methodological quality standards. Furthermore, we recommend further study of the subgroup variables and intervention features that contribute to treatment efficacy.

## INTRODUCTION

An increasing body of empirical research examining the efficacy of psychotherapeutic interventions to help the bereaved is emerging in the scientific literature. In parallel, many efficacy study reviews are also appearing.^1-5^ These reviews represent efforts to bring together available knowledge, in part to provide evidence-based guidance to health care professionals working with bereaved persons at risk for a range of psychological and physical consequences of bereavement. Given the multitude of such research and review work, however, it is now difficult to obtain an overview of this knowledge base. We are left wondering: What do existing reviews actually conclude about the helpfulness of psychotherapeutic interventions? Are reviews aligned in their conclusions? In what ways do their conclusions differ?

The aim of this systematic umbrella review (conducted in accordance with the principles of Pollock and colleagues^6^) is to assess the current state of bereavement-intervention efficacy knowledge from published reviews and meta-analyses. Additionally, we sought to pinpoint commonalities and discrepancies in their conclusions. We systematically assessed the reviews of empirical intervention efficacy studies rather than the empirical studies themselves. Health care professionals often seek out reviews for overviews on expert research; they provide accessible syntheses of the multitude of studies on the topic. We prioritized recent conclusions by limiting our search to reviews examining efficacy and effectiveness of psychotherapeutic grief interventions published after our first review of this literature.^7^ That review was based on a public health framework, covering psychotherapy and empirically tested, methodologically sound quantitative studies. Notably, in our past review, preventive interventions are classified according to three target groups (cf. Caplan^8^). Those classifications are (1) primary: open to all bereaved people (sometimes within specific subgroups, e.g., widowed, bereaved children); (2) secondary: for vulnerable persons (e.g., following traumatic types of death, loss of a child) at high risk for grieving difficulties; and (3) tertiary: aimed at treating complicated/pathological grieving. Our overarching conclusion was that “the more complicated the grief process appears to be or to become, the better the chances of interventions leading to positive results."^7^ We did, however, also identify diverse concerns indicating lack of sound research.

Since our 2001 study, there has not been a comprehensive systematic overview of psychotherapeutic interventions to support bereaved individuals. While expansive, Asgari and colleagues^9^ cover “any reported grief interventions” in their review, not specifically those centered on psychotherapy. An overview of psychotherapeutic intervention is important, notably because prolonged grief disorder (PGD) was recently added to *the Diagnostic and Statistical Manual of Mental Disorders, Fifth Edition, Text Revision* (*DSM-5-TR*) and *the International Classification of Diseases 11th Revision* (*ICD-11*). (Note that we have retained the term “complicated grief” to refer to PGD where it is referred to as such in the cited reviews.) Thus, updated coverage of bereavement intervention efficacy is timely and important for health care professionals to support bereaved individuals.

The aim of this study is to comprehensively review results of quantitative systematic reviews and meta-analyses on the efficacy of bereavement interventions published January 2001–October 2021. We aimed to answer the following research questions:

– Do the reviews published since 2001 indicate that psychotherapeutic interventions for bereaved persons are effective?
– If so, what types of interventions are effective and for whom?
– Has the design and methodological quality of studies improved since 2001?

## METHOD

### Search Strategy

A systematic literature search was conducted in three scientific databases (PsycInfo, Medline, and Web of Science) in May 2018, using the following Boolean search terms for all text: (review OR meta-analysis OR meta analysis OR meta analytic OR meta-analytic) AND (treatment OR treatments OR intervention OR interventions OR care OR therapy OR therapies OR counselling OR counseling) AND (grief OR bereaved OR bereavement). This search was limited to English-language peer-reviewed journal articles published subsequent to Schut and colleagues’^7^ January 2001 review. Reference lists of the included systematic reviews were manually screened for additional eligible reviews. To provide a comprehensive picture of bereavement intervention efficacy pre-COVID-19, an updated search using the same search strategy was conducted for May 2018–October 2021. (Subsequent reviews have been monitored—see Discussion.) An a priori published protocol can be obtained in the PROSPERO register (CRD42018094251). We conducted this review in accordance with Preferred Reporting Items for Systematic Reviews and Meta-Analyses (PRISMA) extension for Scoping Reviews^10^ statement guidelines.

### Inclusion and Exclusion Criteria

Inclusion criteria covered systematic reviews and/or meta-analyses examining the efficacy and effectiveness of psychotherapeutic grief interventions published January 2001–October 2021 in the English language. Psychotherapeutic interventions conducted by trained volunteers and health care professionals were eligible for inclusion. Included reviews focused on symptomatology reduction (e.g., [complicated] grief or depression symptoms) or improvement in well-being (e.g., daily and social functioning) of bereaved people following psychotherapeutic intervention. Due to the broad scope of this systematic review, no restrictions were applied regarding cause of loss or participant demographics.

Reviews were excluded if they (1) focused on medical or psychopharmacological interventions, (2) gave an overview of systematic reviews on bereavement interventions (e.g., Altmaier^11^), (3) were conducted unsystematically (e.g., Hensley^12^), (4) did not primarily focus on bereavement interventions (e.g., Leichsenring and Klein^13^), (5) solely focused on qualitative research, or (6) did not clearly separate qualitative from quantitative findings (e.g., Paraíso and colleagues^14^).

Selection procedure was conducted by two raters (LL, MS) who independently used Covidence. After removing duplicates, titles and abstracts were screened based on inclusion and exclusion criteria. Thereafter, full article texts were screened. Differences between the two raters were resolved through discussion. In the rare case of continued disagreement, another researcher (DvD or HS) evaluated the study and arguments, and suggested a decision for final consensus. The updated search in 2021 followed the same selection and decision procedure, with KA and MS acting as independent raters.

### Quality Assessment

To ensure reliability, all review articles were appraised for quality. Each full-text article that met inclusion criteria was independently examined by two raters (DvD, KA) using the updated Assessment of Multiple Systematic Reviews (AMSTAR-2).^15^ (See Supplement 1 [http://links.lww.com/HRP/A231]). Disagreement was discussed as outlined above (with HS, MM, MS, and/or LL making suggestions in difficult cases).

### Data Extraction

Data were extracted by two authors (KA, MS). To determine interrater agreement, a third rater (LL) independently extracted data from a subset of the articles (*k* = 8). Rating differences were resolved as described above, with DvD, HS, or MM suggesting solutions. (For details of the systematically extracted information, see Supplement 2 [http://links.lww.com/HRP/A232]).

To increase uniformity in the extraction process, predetermined categorical options were agreed upon for some of the items. (A description of these categorical options can be found in Supplement 3 [http://links.lww.com/HRP/A233]). For these items, kappa scores were calculated to determine level of agreement among the raters. For items in which categorical options were not a possibility (e.g., date of publication), data were checked for discrepancies.

Overall, interrater agreement was strong; kappa scores ranged between .38–1.00, with a median score of .83. The lowest score of .38 was found for reviews that classified information according to primary, secondary, or tertiary prevention interventions. For this category, a second rating was conducted by one of the authors (MM) on the remaining reviews (*k* = 25). Any differences resulting from the second rating were resolved by all authors through discussion.

### Intervention Efficacy Analysis

Due to the broad scope and heterogeneous nature of the reviews, a narrative synthesis was undertaken in the data analysis.^16^ This entailed qualitatively summarizing the primary findings on intervention efficacy. First, the descriptive information (publication information, population characteristics, intervention characteristics, outcomes, and research design) was explored. Next, overall conclusions were rated in three categories by strength of evidence for intervention efficacy: positive-unconditional, positive-conditional, and negative-no evidence. Descriptions of these categories are outlined in Table 1.

**Table 1:** Description of Categories for Strength-of-Evidence Analysis

Category	Description
**Positive-unconditional**	Highlights efficacy without constraints
**Positive-conditional**	Some evidence of efficacy, but efficacy is limited by reservations or shortcomings: **Reservations** **Efficacy dependent on:** (a) specific subgroups (e.g., those with prolonged grief) (b) specific times/durations (e.g., only shortly after intervention) (c) specific variables (e.g., for grief, not depression) **Shortcomings** **Efficacy constrained by:** (a) database weaknesses (e.g., few studies; design features) (b) weak effects (e.g., no clinically relevant effects; mixed effects)
**Negative-no evidence**	Insufficient or no evidence of efficacy

## RESULTS

### Study Selection

**Figure 1:**
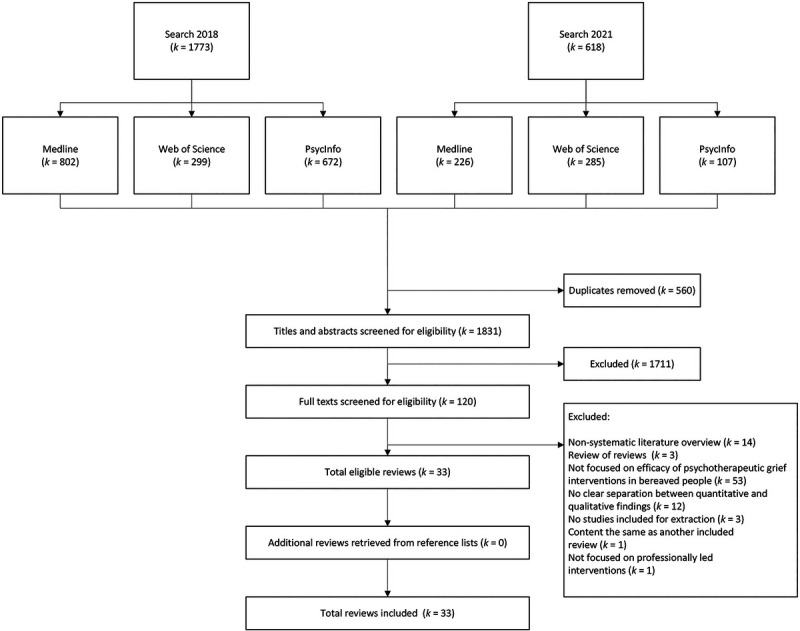
Flowchart of search screening procedure.

In total, 33 systematic reviews and/or meta-analyses were included. For a flowchart of the screening procedure see Figure 1. (For a list of the excluded full-text articles—including reasons for exclusion—see Supplement 4 [http://links.lww.com/HRP/A234]).

### Review Quality

According to the AMSTAR-2 rating system, three reviews were rated high-quality,^17-19^ two moderate,^20,2^ and three low-quality.^3,4,21^ Twenty-five were of critically low-quality.^1,3,5,22-44^ (See Supplement 5 [http://links.lww.com/HRP/A235] for an overview.)

Regarding AMSTAR-2 critical criteria, assessment of risk of bias (ROB) was problematic. Of the 32 reviews that included randomized controlled trials (RCTs), only nine (28.1%) adequately assessed ROB aligned with criteria for RCT implementation. Moreover, of the 25 reviews that included nonrandomized studies of interventions (NRSI), only 4 (16%) adequately assessed ROB in accordance with criteria for NRSI implementation. Generally, there was a lack of focus and clarity. For instance, it was unclear whether outcomes and analyses were selectively reported (e.g., whether authors had prespecified outcomes for assessment in registered protocols or whether they only reported on outcomes that had an effect), whether there was bias in exposure or outcome measurement (e.g., whether assessment occurred in real time or was subject to recall bias), and, in the case of RCTs, whether implementation of a randomization/allocation sequence was adequately concealed. Discussion of ROB on result interpretation was also problematic; only 12 of the 33 reviews (36.4%) gave sufficient attention to this issue.

Furthermore, in the included meta-analyses, appropriate statistical combination methods for results were often lacking; only 4 of the 11 meta-analyses (36.4%) that included RCTs met criteria for appropriate methods, and none that combined the results of NRSIs met criteria. In particular, justification for performing a meta-analysis was infrequently explained (e.g., the meta-analyses often failed to indicate whether studies were compatible in terms of population, intervention, and research design characteristics). Finally, effects of publication bias on results were also inadequate. Only 2 of the 11 meta-analyses (18.2%) assessed publication bias according to AMSTAR-2 guidelines.

### Review Characteristics

A detailed overview of review characteristics can be found in Table 2 (see end of manuscript). (Result patterns are summarized in Supplement 6 [http://links.lww.com/HRP/A236]).

**Table 2:** Review Characteristics and Overall Efficacy of Included Interventions (K = 33)

**First author, date of publication**	**(a) Type of review** **(b) Number of studies exploring intervention efficacy** **(c) Range of publication dates**	**(a) N total** **(b) Age group** **(c) Relationship to the deceased** **(i.e.,** **the participants were the deceased's...) (d) Cause of death**	**(a) Primary (P) secondary (S) & tertiary (T)** **(b) Content of intervention** **(c) Person leading the intervention** **(d) Individual/group**	**(a) Grief-related outcomes assessed (b) Non-grief-related outcomes assessed** **(e.g.,** **PTSD, depression, well-being)**	**(a) Research design** **(b) Type of control group (c) Follow-up reported**	**(a) Overall efficacy of interventions** (b) Category assignment*
Curtis & Newman (2001)^27^	(a) Narrative-synthesis (b) 9 (c) 1992-1999	(a) Not specified (b) Children (c) Varied (d) Varied	(a) P (b) Varied (c) Not specified (d) Not specified	(a) Not specified (b) Yes	(a) Varied (b) Not specified (c) Not specified	(a) “There is a small amount of quantitative evidence that community interventions benefit parents and children within a bereaved family, but evidence is too weak to make judgments about the relative effectiveness of different models of community-based interventions.” (b) Positive-conditional: database weaknesses
Rowa-Dewar (2002)^40^	(a) Narrative-synthesis (b) 3 (c) 1994-2000	(a) Not specified (b) Adults (c) Parent (d) Varied	(a) S (b) Varied (c) Varied (d) Not specified	(a) Yes (b) Yes	(a) Controlled studies (b) Varied (c) Some studies	(a) “No overall benefit for the interventions was shown. However, for highly distressed mothers, psychological symptoms and marital dysfunction were significantly reduced.” (b) Positive-conditional: some subgroups
Schut & Stroebe (2005)^41^	(a) Narrative-synthesis (b) 7 (c) 1999-2004	(a) Not specified (b) Not specified (c) Varied (d) Varied	(a) Combination of P, S, & T (b) Not specified (c) Not specified (d) Both individual & group	(a) Yes (b) Yes	(a) Controlled studies (b) Varied (c) Not specified	(a) “Routine intervention for bereavement has not received support from quantitative evaluations of its effectiveness and is therefore not empirically based . . . Intervention is more effective for those with more complicated forms of grief.” (b) Positive-conditional: some subgroups
Currier et al. (2007)^1^	(a) Meta-analysis (b) 13 (c) 1987-2003	(a) Not specified (b) Children (c) Varied (d) Varied	(a) Combination of P, S, & T (b) Psychoeducation (c) Varied (d) Both individual & group	(a) Yes (b) Yes	(a) Controlled studies (b) No treatment (c) Some studies	(a) “Child grief interventions do not appear to generate the positive outcomes of other professional psychotherapeutic interventions. However, studies that intervened in a time-sensitive manner and those that implemented specific selection criteria produced better outcomes than investigations that did not attend to these factors.” (b) Positive-conditional: some subgroups
Currier et al. (2008)^25^	(a) Meta-analysis (b) 61 (c) 1975-2010	(a) Not specified (b) Varied (c) Varied (d) Varied	(a) Combination of P, S, & T (b) Varied (c) Varied (d) Both individual & group	(a) Yes (b) Yes	(a) Controlled studies (b) No treatment (c) Some studies	(a) “Overall, analyses showed that interventions had a small effect at post-treatment but no statistically significant benefit at follow-up. However, interventions that exclusively targeted grievers displaying marked difficulties adapting to loss had outcomes that compare favorably with psychotherapies for other difficulties.” (b) Positive-conditional: some subgroups
McDaid et al. (2008)^18^	(a) Narrative-synthesis (b) 8 (c) 1992-2007	(a) Not specified (b) Varied (c) Varied (d) Suicide	(a) S (b) Varied (c) Varied (d) Not specified	(a) Yes (b) Yes	(a) Controlled studies (b) Varied (c) Some studies	(a) “Although there is evidence of some benefit from interventions for people bereaved by suicide, this is not robust. Further methodologically sound evidence is required to confirm whether interventions are helpful and, if so, for whom.” (b) Positive-conditional: database weaknesses
Rosner et al. (2010)^39^	(a) Meta-analysis (b) 27 (c) 1982-2005	(a) 1,073 (b) Children (c) Varied (d) Varied	(a) Combination of P, S, & T (b) Varied (c) Not specified (d) Both individual & group	(a) Yes (b) Yes	(a) Varied (b) Varied (c) Some studies	(a) “The omnibus effect sizes in our meta-analysis of controlled studies and uncontrolled studies are 0.35 and 0.49, respectively. Our results thus indicate a small to moderate treatment effect.” (b) Positive-unconditional
Currier et al. (2010)^26^	(a) Meta-analysis (b) 11 (c) 1981-2007	(a) Not specified (b) Adults (c) Varied (d) Varied	(a) S & T (b) CBT (c) Not specified (d) Both individual & group	(a) Yes (b) Yes	(a) Controlled studies (b) Varied (c) Some studies	(a) "Compared to no-treatment control groups, CBT-based interventions were beneficial immediately after intervention (d = 0.38) but did not yield statistically significant overall effects at follow-up (d = 0.18)." (b) Positive-conditional: some times/durations
Wittouck et al. (2011)^5^	(a) Meta-analysis (b) 14 (c) 1988-2007	(a) 1655 (b) Adults (c) Varied (d) Varied	(a) Combination of P, S, & T (b) Varied (c) Varied (d) Both individual & group	(a) Yes (b) No	(a) RCT (b) Varied (c) Some studies	(a) “Treatment interventions can effectively diminish complicated grief symptoms. Preventive interventions, on the other hand, do not appear to be effective.” (b) Positive-conditional: some subgroups
Murphy et al. (2012)^19^	(a) Narrative-synthesis (b) 6 (c) 1996-2009	(a) 1001 (b) Adults (c) Parent (d) Perinatal loss	(a) S (b) Varied (c) Varied (d) Individual	(a) Yes (b) Yes	(a) RCT (b) Varied (c) Some studies	(a) “Evidence is insufficient to demonstrate that psychological support such as counseling is effective post-miscarriage. Further trials should be good quality, adequately-powered using standardised interventions and outcome measures at specific time points.” (b) Negative-no evidence
Hefren & Thyer (2012)^32^	(a) Narrative-synthesis (b) 7 (c) 1978-2001	(a) Not specified (b) Adults (c) Not specified (d) Not specified	(a) T (b) Guided mourning (c) Varied (d) Not specified	(a) Yes (b) Yes	(a) Varied (b) Varied (c) Some studies	(a) “While it appears that this intervention is effective, it must be carefully understood… there are specific circumstances when the evidence is not convincing, and it appears that there are very specific circumstances where GM is an appropriate course of treatment and specific circumstances where it would not be appropriate.” (b) Positive-conditional: some subgroups
Nagy & Szamosköz (2013)^37^	(a) Meta-analysis (b) 11 (c) 2001-2011	(a) 541 (b) Adults (c) Varied (d) Varied	(a) T (b) CBT (c) Varied (d) Both individual & group	(a) Yes (b) Yes	(a) Not specified (b) Varied (c) Not specified	(a) “Our results show that cognitive behavioral interventions have no significant effect neither on complicated grief symptoms or co-morbid symptoms, neither at post-treatment or at follow-up.” (b) Negative-no evidence
Nseir & Larkey (2013)^38^	(a) Narrative-synthesis (b) 7 (c) 1993-2010	(a) Not specified (b) Adults (c) Spouse (d) Varied	(a) P (b) Varied (c) Varied (d) Both individual & group	(a) Yes (b) Yes	(a) Controlled studies (b) Varied (c) Some studies	(a) “All intervention types showed improvements in grief-related outcomes, but statistical significance of major measures between interventions and controls was absent in all but one study using a mind-body intervention.” (b) Positive-conditional: weak effects
Jones et al. (2015)^33^	(a) Narrative-synthesis (b) 20 (c) 1982-2012	(a) Not specified (b) Not specified (c) Parent (d) Perinatal loss	(a) S & T (b) Varied (c) Varied (d) Both individual & group	(a) Yes (b) Yes	(a) Varied (b) Varied (c) Some studies	(a) “This review offers preliminary support for offering individual psychological interventions for mothers displaying high rates of grief and mental health symptoms. However, with the evidence base at such an early stage of development, clinicians and services are recommended to use non-specific psychological formulation skills in the formation of individualised psychological treatments.“ (b) Positive-conditional: database weaknesses
Endo et al. (2015)^2^	(a) Narrative-synthesis (b) 8 (c) 1979-2011	(a) Not specified (b) Varied (c) Parent (d) Varied	(a) S (b) Varied (c) Varied (d) Both individual & group	(a) Yes (b) Yes	(a) RCT (b) Varied (c) Some studies	(a) “Most intervention trials showed some effect on participants in at least one outcome measure. However, we identified many severe methodological issues and outcome sets in these trials.” (b) Positive-conditional: database weaknesses
Waller et al. (2016)^21^	(a) Narrative-synthesis (b) 45 (19 primary interventions) (c) 2001-2014	(a) 2,685 individuals & 257 families (b) Adults (c) Varied (d) Varied	(a) Combination of P, S, & T (b) Varied (c) Not specified (d) Both individual & group	(a) Yes (b) Yes	(a) Varied (b) Varied (c) Some studies	(a) “The three studies that met all criteria showed mixed effectiveness . . . Nevertheless, these findings indicate that both complicated grief therapy and family-focused therapy show potential as effective interventions for alleviating grief symptoms and should be further explored and replicated including usual care groups.” (b) Positive-conditional: weak effects
Enez (2017)^29^	(a) Narrative-synthesis (b) 21 (c) 2001-2016	(a) Not specified (b) Not specified (c) Varied (d) Varied	(a) T (b) Varied (c) Varied (d) Both individual & group	(a) Yes (b) Yes	(a) Varied (b) Varied (c) Some studies	(a) “This systematic review found some evidence to support the effectiveness of psychotherapy-based interventions on patients' recovering from CG. The included studies reported positive outcomes for these interventions and indicated its effectiveness in both the short- and long-term.” (b) Positive-unconditional
Journot-Reverbel et al. (2017)^34^	(a) Narrative-synthesis (b) 2 (c) 1975-2016	(a) Not specified (b) Children (c) Varied (d) Suicide	(a) S (b) Supportive therapy (c) Not specified (d) Group	(a) No (b) Yes	(a) Varied (b) Only one of the two studies had a control group that compared “a support bereavement group intervention vs. a bereavement group without intervention.” (c) No studies	(a) “We only found two articles specifically targeting children or adolescents: both of them seemed to show some efficacy in reducing some psychosocial variables (anxiety, depression…) in suicide-bereaved children but results were limited by methodological problems.” (b) Positive-conditional: database weaknesses
Linde et al. (2017)^4^	(a) Narrative-synthesis (b) 7 (c) 1992-2014	(a) Not specified (b) Varied (c) Varied (d) Suicide	(a) S (b) Varied (c) Varied (d) Both individual & group	(a) Yes (b) No	(a) Varied (b) Varied (c) Some studies	(a) “… preliminary results indicate some positive effects of interventions in reducing grief intensity and suicide-specific aspects of grief. Study results regarding complicated grief are less promising.” (b) Positive-conditional: some variables
Bergman et al. (2017)^24^	(a) Narrative-synthesis (b) 17 (c) 1985-2015	(a) Not specified (b) Children (c) Child (d) Varied	(a) Combination of P, S, & T (b) Varied (c) Varied (d) Both individual & group	(a) Yes (b) Yes	(a) Varied (b) Varied (c) Some studies	(a) “The results indicate that relatively brief interventions can prevent children from developing more severe problems after the loss of a parent, such as traumatic grief and mental health problems. Studies have shown positive effects for both children’s and remaining caregiver’s health.” (b) Positive-unconditional
Andriessen et al. (2019)^17^	(a) Narrative-synthesis (b) 11 (c) 1984-2018	(a) Not specified (b) Varied (c) Varied (d) Suicide	(a) S & T (b) Varied (c) Varied (d) Both individual & group	(a) Yes (b) Yes	(a) Controlled studies (b) Varied (c) Some studies	(a) “While there was some evidence of the effectiveness of interventions for uncomplicated grief, evidence of the effectiveness of complicated grief interventions was lacking.” (b) Positive-conditional: some variables
Johannsen et al. (2019)^3^	(a) Meta-analysis (b) 31 (c) 1992-2017	(a) 4760 (b) Adults (c) Varied (d) Varied	(a) Combination of P, S, & T (b) Varied (c) Not specified (d) Both individual & group	(a) Yes (b) Yes	(a) RCT (b) Varied (c) Some studies	(a) “Taken together, the results indicate that while psychological interventions for grief may be efficacious even when adjusting for publications bias, the effects are small (Hedges’ g: 0.41–0,45), thereby not meeting the criteria suggested for a clinically relevant effect, which has previously been defined as 0.50."^47^ (b) Positive-conditional: weak effects
Alves-Costa et al. (2019)^22^	(a) Narrative-synthesis (b) 7 (c) 2001-2018	(a) Not specified (b) Varied (c) Varied (d) Homicide	(a) S & T (b) Varied (c) Varied (d) Both individual & group	(a) Yes (b) Yes	(a) Varied (b) Not specified (c) Some studies	(a) “Overall, symptoms of PTSD, CG, and depression decreased significantly postintervention. Sustained improvements were reported for PTSD and depressive symptoms at the follow-up measurements.” (b) Positive-unconditional
Andriessen et al. (2019)^20^	(a) Narrative-synthesis (b) 8 (c) 2014-2019	(a) Not specified (b) Varied (c) Varied (d) Suicide	(a) S & T (b) Varied (c) Varied (d) Both individual & group	(a) Yes (b) Yes	(a) Varied (b) Varied (c) Some studies	(a) “This review found limited evidence of effectiveness of postvention interventions and service delivery, mainly due to a relative shortage of research, particularly high-quality research involving control groups.” (b) Positive-conditional: database weaknesses
Fiore (2019)^31^	(a) Narrative-synthesis (b) 12 (c) 2005-2016	(a) Not specified (b) Adults (c) Parent (d) Not specified	(a) Combination of P, S, & T (b) Interventions based on Dual Process Model framework (c) Not specified (d) Both individual & group	(a) Yes (b) Yes	(a) Varied (b) Varied (c) Not specified	(a) “…interventions based upon the DPM may be effective for treatment of grief, stress and coping. Despite positive support, more research is needed to understand both the model and how interventions based on the DPM impact the bereavement process.” (b) Positive-conditional: database weaknesses
Mason et al. (2020)^36^	(a) Narrative-synthesis (b) 16 (c) 2009-2018	(a) 4,843 (b) Adults (c) Varied (d) Not specified	(a) T (b) Varied (c) Not specified (d) Both individual & group	(a) Yes (b) Yes	(a) Varied (b) Varied (c) Some studies	(a) “This review of the literature . . . identified potential therapies for family caregivers experiencing CG. Individual counseling and the use of support groups are the primary methods of providing bereavement services that are supported by research evidence.” (b) Positive-unconditional
Maass et al. (2020)^35^	(a) Meta-analysis (b) 14 (c) 1996- 2019	(a) 1,519 (b) Adults (c) Varied (d) Varied	(a) Combination of P, S, & T (b) Varied (c) Varied (d) Group	(a) Yes (b) Yes	(a) RCT (b) Varied (c) Some studies	(a) “The general result from this meta-analysis is that the current state of evidence on bereavement groups is rather weak, because their effects were, in comparison with control groups, only small post-treatment and non-significant at follow-up.” (b) Positive-conditional: weak effects
Bagheri et al. (2020)^23^	(a) Meta-analysis (b) 7 (c) 2006-2017	(a) 918 (b) Not specified (c) Parent (d) Perinatal loss	(a) S (b) Varied (c) Not specified (d) Not specified	(a) Yes (b) Yes	(a) Varied (b) Varied (c) Some studies	(a) “This systematic review found psychotherapy-based interventions are effective in post-abortion grief treatment but; we found psychotherapy-based interventions are somewhat effective in short-term postabortion grief and it has a better effect on long-term grief.” (b) Positive-conditional: some times/durations
Wagner et al. (2020)^43^	(a) Meta-analysis (b) 7 (c) 2006-2015	(a) 1257 (b) Adults (c) Varied (d) Varied	(a) Combination of P, S, & T (b) CBT (c) Not specified (d) Not specified	(a) Yes (b) Yes	(a) RCT (b) Waitlist (c) Some studies	(a) “… While online treatment is largely effective in treating PTSD symptoms, its effect on grief symptoms themselves is comparably lower, although moderate and stable over time. The effects on depression, however, are less clear and tend to be small.” (b) Positive-conditional: some variables
de López et al. (2020)^28^	(a) Narrative-synthesis (b) 8 (c) 1985-2010	(a) Not specified (b) Children (c) Varied (d) Not specified (only clear for one study that cause was suicide)	(a) S & T (b) Not specified (c) Not specified (d) Both individual & group	(a) Yes (b) Yes	(a) Controlled studies (b) Varied (c) Some studies	(a) “The overall result of our review shows that controlled treatment studies for children and adolescents suffering from prolonged grief remain relatively few, with just two studies measuring grief as an individual outcome measure, but these showed promising short-term (Tonkins et al.) and long-term (Sandler et al.) results from the treatment of complicated grief in bereaved children.” (b) Positive-conditional: database weaknesses
Shaohua & Shorey (2021)^42^	(a) Meta-analysis & narrative-synthesis (b) 17 (c) 1982-2019	(a) 2065 (b) Varied (c) Parent (d) Perinatal loss	(a) S (b) Varied (c) Varied (d) Both individual & group	(a) Yes (b) Yes	(a) RCT (b) Varied (c) Some studies	(a) “This review shows that despite constraints from very poor overall quality, psychosocial interventions are effective in reducing depression, anxiety, and grief amongst parents with perinatal loss.” (b) Positive-conditional: database weaknesses
Wojtkowiak et al. (2021)^44^	(a) Narrative-synthesis (b) 22 (c) 2009-2019	(a) Not specified (b) Varied (c) Varied (d) Varied	(a) Combination of P, S, & T (b) Interventions all included ritual elements (c) Not specified (d) Both individual & group	(a) Yes (b) Yes	(a) Varied (b) Not specified (c) Some studies	(a) “Almost all studies show significant effects of the grief treatment, trauma and related symptoms. However, the effects are mostly measured for the entire treatment and not separately for the ritual intervention.” (b) Positive-unconditional
Fernández-Férez et al. (2021)^30^	(a) Narrative-synthesis (b) 4 (c) 2017-2019	(a) Not specified (b) Adults (c) Parent (d) Perinatal loss	(a) S (b) Varied (c) Varied (d) Both individual & group	(a) Yes (b) Yes	(a) Controlled (b) Not specified (c) Not specified	(a) “The interventions that were analyzed positively improve psychological self-concept and role functions, as well as mutual commitment, depression, post-traumatic stress and symptoms of grief. These interventions are effective if they are carried out both before perinatal loss and after it has occurred.” (b) Positive-unconditional

**Note:**

*Categories:

**Positive-unconditional:** highlights the efficacy without constraints.

**Positive-conditional:** some evidence of efficacy, but efficacy is limited by reservations or shortcomings (specific subgroups [e.g., those with prolonged grief], specific times/durations [e.g., only shortly after intervention], specific variables [e.g., for grief, not depression], database weaknesses [e.g., too few studies, design features], weak effects [e.g., no clinically relevant effects, mixed effects])

**Negative-no evidence:** insufficient or no evidence of efficacy

### Intervention Efficacy

### Positive-unconditional

Seven of the 33 reviews were rated positive-unconditional, indicating conclusions that strongly support the efficacy of bereavement interventions.^22,24,29,30,36,39,44^ (Characteristics of these reviews are summarized in Supplement 6.)

Effects were reported on a wide variety of grief- and non-grief-related outcomes. Six of the seven reviews narratively synthesized results, reporting significant effects on measures such as grief (and complicated grief) symptoms,^22,24,29,30,36,44^ posttraumatic stress disorder (PTSD),^22,24,30^ depression,^22,24,29,30,36,44^ anxiety,^29,36^ psychological self-concept and role functions,^30^ and mutual commitment.^30^ Furthermore, the meta-analysis by Rosner and colleagues,^39^ which focused on child and adolescent interventions, found small (Hedges *g* = 0.35) to moderate overall effects (Hedges *g* = 0.49) on outcomes for grief, depression, anxiety, PTSD, social adjustment, well-being, and somatic symptoms for controlled and uncontrolled studies respectively.

## Positive-conditional

Twenty-four of the 33 reviews were categorized as positive-conditional.^1-5,17,18,20,21,23,25-28,31,32-35,38,40-43^ While they generally found support for bereavement interventions, they also drew attention to reservations (11 of the 24 reviews) or shortcomings (13 of the 24 reviews) limiting effectiveness. (See Supplement 6 for a summary of review characteristics.)

## Reservations

### Specific subgroups

Six of the 24 positive-conditional reviews highlighted that efficacy appears dependent on specific sugroups.^1,5,25,32,40,41^ The majority drew attention to the relationship between initial distress levels or symptom severity on intervention efficacy. For example, in Rowa-Dewar’s^40^ review focusing on parental bereavement support, no overall benefits for bereavement interventions were found. The author did, however, report improvement on a number of outcomes (e.g., mental distress, posttraumatic distress, loss accommodation) for a subgroup of mothers and fathers who were distressed or at high risk for poor adjustment. Likewise, Schut and Stroebe^41^ also concluded that bereavement interventions were generally more effective for individuals with grieving process complications; though, the authors also drew attention to improved outcomes for primary prevention interventions compared to their 2001 review.

These findings were further supported by Wittouck and colleagues,^5^ who included RCTs exploring intervention efficacy for the prevention and treatment of complicated grief. They concluded that treatment interventions were effective in reducing complicated grief (pooled *SMD* = -0.53 [post-intervention], pooled *SMD* = -1.38 [follow-up]). Preventive interventions, which generally targeted bereaved people, did not, however, appear effective (pooled *SMD* = -0.03 [post-intervention], pooled *SMD* = 0.13 [follow-up]). Similarly, Currier and colleagues^25^ computed mean effect sizes for a variety of outcome measures, such as depression, relational functioning, and well-being. They found no significant effects for universal preventive interventions (those targeting all bereaved individuals) at post-treatment or follow-up. In contrast, they reported a small within-group effect for selective interventions (those targeting bereaved individuals at high risk for developing symptoms of distress) at post-treatment (*d* = 0.14). Further, they found a moderate within-group effect for indicated interventions (those targeting bereaved individuals who display clinically significant difficulties) at both post-treatment (*d* = 0.53) and follow-up (*d* = 0.58).

### Specific times/durations

Two of the 24 positive-conditional reviews highlighted the influence of assessment time on intervention efficacy.^23,26^ Currier and colleagues^26^ explored cognitive behavioral therapy (CBT) intervention effects on grief, depression, anxiety, trauma, and general distress. Compared to no-treatment control groups, CBT interventions generally appeared effective immediately after treatment (*d =* 0.38). Notably, however, there were no significant effects reported at follow-up (*d* = 0.18), which occurred, on average, eight months after intervention.

In contrast, Bagheri and colleagues^23^ investigated post-abortion grief interventions. They appeared to have a small effect directly after treatment, with continued improvement three to four months later. Neither the post-intervention effects (*SMD* = −0.03, *p* = 0.87) nor the follow-up effects (*SMD* = -0.2, *p* = 0.19), however, achieved significance.

### Specific variables

Three of the 24 positive-conditional reviews further highlighted the efficacy differences among the various outcome variables measured.^17,4,43^ Linde and colleagues^4^ investigated grief interventions for those bereaved by suicide. While interventions appeared effective in reducing grief intensity and suicide-specific aspects of grief, abatement of complicated grief was less effective. Notably, only one of the two studies that assessed complicated grief found effects for prevention, and this finding was only for a subgroup with high suicidal ideation. Similarly, Andriessen and colleagues^17^ explored interventions for suicide-related bereavement. They found some positive intervention outcomes for uncomplicated grief, but not for complicated grief.

Furthermore, Wagner and colleagues^43^ explored the efficacy of web-based, controlled-study interventions on grief- and non-grief-related outcomes, and found efficacy differences between these outcomes. Specifically, they found large (between-group) effects for PTSD (Hedges *g* = .86), moderate effects for grief (Hedges *g* = .54), and only small effects for depression (Hedges *g* = .44). Higher effect sizes were, however, found for depression interventions that included more personal therapeutic feedback (e.g., individualized, writing assignments) compared to those with informative feedback (e.g., general, about the program/homework) or no feedback (*B* = 0.20, 95% CI [0.002, 0.39]).

## Shortcomings

### Database weaknesses

Nine of the 24 positive-conditional reviews drew attention to methodological limitations of empirical studies in their conclusions.^2,18,20,27,28,31,33,34,42^ While they found effects on some outcome measures, such as complicated grief, suicidality, anxiety, PTSD, depression, and personal growth, methodological issues prevented drawing strong supportive conclusions for bereavement interventions.

In particular, these reviews highlighted the scarcity of studies exploring intervention efficacy,^20,28,31,34^ as well as other methodological limitations, including a lack of control groups,^20,34^ nonrandomized allocation,^18,34^ small sample sizes,^2,18,27,33,34,42^ high attrition,^27,34^ and lack of long-term follow-up measures.^27,34^ Furthermore, the reviews also highlighted the heterogeneous outcome measures used by the different studies,^2,33^ making it difficult to draw comparisons among the studies, as well as a lack of clarity in the methods and outcomes used.^2,33^

### Weak effects

Four of the 24 positive-conditional reviews focused on weak effects (e.g., no clinically relevant effects, mixed effects) found for bereavement interventions.^3,21,35,38^ Two of the reviews highlighted the mixed effects found among the studies. For example, Nseir and Larkey’s^38^ narratively synthesized review explored spousal bereavement interventions. They noted that all studies showed some improvement compared to baseline, but significant differences between intervention and control group were only achieved in one study (a mind-body intervention^45,46^). This intervention specifically found effects on measures of grief, stress, depression, and life satisfaction. Similarly, Waller and colleagues^21^ explored grief counseling interventions for adults in their narrative synthesis. They found some support for complicated grief therapy relative to interpersonal therapy, but effects for family-focused therapy were less clear; only one of the two studies focused on this approach reported improvements.

The other two (meta-analytic) reviews drew attention to the small effects found for bereavement interventions on both grief- and non-grief-related outcomes. Maass and colleagues^35^ explored bereavement group efficacy. While they found some between-group effects post-intervention, these effects were small for both grief (Hedges *g* = 0.33) and depression (Hedges *g* = 0.22). Furthermore, no significant effects for either of these outcomes were found at follow-up. Likewise, Johannsen and colleagues^3^ explored RCTs tailored to bereaved adults. They concluded that grief outcome effects at post-intervention (Hedges *g* = 0.41) and follow-up (Hedges *g* = 0.45) were not large enough to meet criteria for a clinically relevant effect size of 0.50, as defined by Norman and colleagues.^47^

### Negative-No Evidence

Finally, 2 of the total 33 reviews were deemed negative-no evidence.^19,37^ These reviews found insufficient or no evidence of bereavement intervention effectiveness. (Characteristics of these reviews are detailed in Supplement 6.) Both of these reviews explored efficacy on a number of outcome variables, including complicated grief, anxiety, depression, emotional disturbance, self-esteem, and isolation. They found limited effects for all of these measures. In their meta-analysis, Nagy and Szamosközi^37^ found no significant effects for complicated grief (post-intervention: *d* = .27, 95% CI [-.22–.77]; follow-up: *d* = .22, 95% CI [-.33–.77]), depression (post-intervention: *d* = -.53, 95% CI [-.82–.15]; follow-up: *d* = -.18, 95% CI [-.58–.22]), or anxiety (post-intervention: *d* = -.36, 95% CI [-.92–.19]). Similarly, Murphy and colleagues’ narrative synthesis^19^ found limited support for interventions focusing on miscarriage loss. Of the six studies included in this review, only one trial reported statistically significant effects. These effects were found on subscales of the Impact of Miscarriage Scale,^48^ a tool developed by the study’s author, with no significant effects found on the standardized measures employed in the study (e.g., Rosenberg Self-Esteem Scale^49^).

## DISCUSSION

This systematic umbrella review aimed to provide an overview of psychotherapeutic intervention efficacy for bereaved individuals. Given the negative consequences associated with bereavement (see Stroebe and colleagues^50^), and the recent addition of PGD to the *DSM-5-TR* and *ICD-11*, this review was deemed timely and worthy, particularly as guidance for health care professionals supporting bereaved individuals.

### Review and Empirical Study Quality

While review findings indicate some support for bereavement intervention value (7 reviews indicated strong support; 24 reviews found partial support; 2 reviews indicated insufficient support), the overall low quality of reviews is worrying. Such low quality is especially concerning since our findings suggest a possible association between review quality and conclusions. In particular, higher-quality reviews seem to draw more cautious conclusions.

Furthermore, the empirical studies included in the assessed reviews were subject to serious methodological problems, further limiting our ability to reach clear conclusions about intervention efficacy. Of particular note is the lack of long-term follow-up assessments in empirical studies. For example, duration of follow-up assessment was less than a year for the studies included in reviews that calculated the average length of follow-up.^25,26,3^ Furthermore, a lack of control groups in some studies may have influenced conclusions about intervention efficacy. Indeed, the majority of reviews that expressed reservation about interventions included controlled studies (*k* = 15). Conversely, of reviews that indicated strong support, only one focused on controlled designs; the rest (*k* = 6) included varied designs.

Thus, empirical studies and systematic reviews must adhere to methodological quality standards to provide reliable conclusions about intervention efficacy for bereaved individuals. Logically, not all included articles adhered to AMSTAR-2 criteria since some (e.g.,^27,40^) were published before the original criteria were developed (AMSTAR in 2007 and AMSTAR-2 in 2017). We urge future review authors to follow criteria for such quality rating tools as the PRISMA checklist^51^ and AMSTAR-2 rating system.^15^ Furthermore, future research needs to carefully consider RCT implementation (e.g., with a waitlist control condition to exclude extraneous variables; to enable efficacy comparison of different treatments). And while length of follow-up assessment will likely depend on various factors, researchers should consider implementing long-term follow-up periods of at least a year (preferably longer) to obtain comprehensive understanding of longer-term treatment effects.

### Directions for Future Research

Despite the overall low quality of reviews and methodological limitations of studies, certain findings emerged from the current review that provide important directions for future research.

### Initial distress level

A critically important point that emerged pertains to assessment of initial distress level or symptom severity (notably^5,25,40,41^). These reviews generally indicate more positive outcomes when individuals with higher levels of distress or grieving process complications are targeted. As suggested by Schut and colleagues,^41^ however, there may be specific circumstances contributing to improved primary intervention outcomes, such as in-reaching strategies (where requests for help come from the bereaved individuals), or later intervention (i.e., after several months/years of bereavement). Although, given the shortage of research (only two reviews focus on primary prevention interventions^26,37^), arriving at firm conclusions about primary prevention intervention efficacy is difficult.

Further examining primary prevention efforts is of paramount importance: (1) Other mental health fields have demonstrated the benefits of studying primary prevention interventions, showing reduced societal costs and decreased distress symptoms, such as depression, aggression, and conduct problems.^52^ (2) There are specific needs inherent to primary prevention interventions: First, while individuals with high distress levels or complications may require psychotherapy or counseling by trained mental health professionals, providing practical help and psychoeducation driven by empirically based theory may be more appropriate for individuals without complications.^53^ Second, outcome measures used to assess primary prevention intervention effectiveness need special evaluation. Assessing severe symptomology in the generally bereaved population, for example, is not always useful given that this population may not score high on these measures in the first place. Future intervention studies should, therefore, carefully consider outcome measures appropriate for the prevention type. General measures of distress and mental health functioning (e.g., the Kessler Psychological Distress Scale^54^ and the General Health Questionnaire^55^) may be deemed more appropriate for interventions that target the generally bereaved than established outcome measures for grief complications (e.g.,^56,57^).

### Time since loss, gender, and age

While the included reviews predominantly drew attention to initial distress levels in their conclusions, other subgroup variables were also highlighted that may be important for intervention efficacy, notably:

– Duration of bereavement: Currier and colleagues^1^ drew attention to nonsignificant trends suggesting improved outcomes for children who received an intervention closer to a loss than for those who received the same intervention after a longer duration. Other reviews that focus on children, adolescents,^39^ and adults,^3^ however, indicate that larger effects were found for studies with a longer duration between bereavement and intervention. As highlighted by Rosner and colleagues,^39^ the influence of time may be attributed to an individual’s need for intervention; those with a longer time between loss and intervention may demonstrate a more complicated grieving process and, thereby, have greater need for treatment. Our understanding of how time since loss may affect intervention efficacy is limited. Thus, we need further insight into how this variable may influence intervention outcomes.
– Gender: Some reviews suggest better intervention outcomes for girls and women on both grief-related and non-grief-related measures.^22,24,40,41^ Time of assessment may be an important aspect when considering gender. For example, Rowa-Dewar’s^40^ narrative synthesis found that while distressed mothers appear to benefit from intervention at 6 months follow-up, this is not the case for fathers. Effects were, however, found at 15 months follow-up for high-risk fathers in one study included in their review,^58^ suggesting that gender plays a role in symptom progress following intervention. Other factors, such as intervention content, may also be important. For example, Schut and colleagues^59^ indicate that problem-focused interventions are more effective for women, while emotion-focused interventions are more effective for men. They suggest that men appear to be generally more inclined toward problem-focused coping and women toward emotion-focused coping. An intervention strategy counter to one’s natural tendency may allow for an individual to fulfill a coping deficit, thereby decreasing distress.
– Age: Three reviews^28,37,3^ indicate that age may affect efficacy of bereavement intervention. Only one of these reviews, however, provides clarity on the direction of this effect, finding larger effects for older than younger adults on the grief outcome at follow-up.^3^

### Intervention characteristics

Despite a lack of focus on intervention features, a few characteristics emerged that may help us better understand intervention efficacy. Improved efficacy seems to be associated with interventions that (1) are led by trained facilitators,^17,20^ (2) involve the social environments of the bereaved,^17,29^ (3) provide individual sessions (rather than group),^3^ and (4) have multiple sessions.^17,31,38,39,42,43^

These characteristics may not be beneficial in all situations however. For example, Shaohua and Shorey’s review,^42^ which explores psychosocial interventions for perinatal loss, indicates that while multisession interventions appear more effective in reducing depression for parents who suffer a miscarriage, this is not the case with other types of perinatal loss. This finding suggests that taking account of such factors as type of loss may be important for understanding how number of sessions influences outcomes. Johannsen and colleagues^3^ explored psychological interventions for bereaved adults, finding greater benefit for individual (rather than group) sessions for grief reduction. The authors, however, highlight that these findings are at odds with other research, suggesting benefits of the group format.^60,61^ These contrasting findings show the need to explore the specific circumstances for which such intervention features may be desirable.

## Review Limitations

This umbrella review is not without limitations. First, while the locations of the review articles’ first authors encompass a variety of English-speaking countries (e.g., United Kingdom, United States, Australia) and non-English-speaking countries (e.g., Iran, Sweden, Japan), the reviews were confined to those published in the English language. Thus, it is possible that relevant reviews published in other languages were missed.

Second, while our first literature search was conducted in May 2018, and subsequently in October 2021, we were unable to conduct a third literature search before publication submission. Our research involved a comprehensive quality analysis (conducted by two independent raters) as well as an in-depth extraction process (multiple categories extracted). A third search would, therefore, replicate a formal process that takes months and requires updating the results. As we based our results on the conclusions of review articles, however, it is unlikely that a third search would alter our conclusions significantly. Implementing primary studies, as well as review articles that assess these primary studies, is a time-consuming process.^62^ Thus, it may take considerable time before the databases—and thus, conclusions—change substantially. Indeed, while a handful of reviews meeting our inclusion criteria have been published subsequent to October 2021 (e.g., Breen and colleagues,^63^ Hanauer and colleagues,^64^ Zuelke and colleagues^65^), these reviews, like those included in our review, generally include intervention studies published before 2019 (86% [92] of studies were published before 2019 in Breen and colleagues^63^, 62% [84] in Hanauer and colleagues'^64^ review, and 100% in Zuelke and colleagues^65^). Therefore, conclusions are similar to ours. For example, these reviews also found some support for bereavement intervention efficacy on outcomes such as grief,^64,65^ anxiety,^63^ PTSD,^64,65^ and depression.^63-65^ Even so, it is possible that surveying interventions implemented subsequent to our review may result in additional relevant insights (e.g., following COVID-19, intervention needs/efficacy could drastically change, justifying separate analysis). It is, therefore, recommended that future meta-reviews focus on assessing more recently implemented interventions for a more comprehensive understanding of bereavement intervention efficacy.

Third, a key problem with collating the findings of other reviews is potential overlap in the empirical studies of different review articles.^66^ Indeed, in the current review, 31.3% of empirical studies are discussed in two or more reviews, 17 of which (5.99%) are discussed in 5 or more reviews. Pfeffer and colleagues^67^ is of particular note. This study, which explores a group intervention for children bereaved by suicide, was included in eight different review articles.^1,17,18,25,28,34,39,41^ Such overlap may result in repetition, rather than new knowledge, potentially giving disproportionate weight to particular studies^66^ and over- or underestimation of effects shown.

Fourth, while categorizing review results according to granular subgroup/intervention characteristics could be useful, the reviews often included combinations of these characteristics. Therefore, broadly categorizing primary conclusions into three categories (i.e., positive-unconditional, positive-conditional, negative-no evidence) was most fitting for our data. This categorization system allowed us to draw conclusions regarding the overall efficacy of bereavement interventions, but not to address all of our original research aims; more nuanced documentation/overview was not possible. For example, we noted general conclusions that initial level of distress influences efficacy. Additional characteristics discussed in many reviews (i.e., time since loss, gender, age), were not highlighted in our general conclusions. (We draw attention to this limitation in our discussion.) Broad categorization is a first step. As more nuanced information becomes available, extending these categories to include additional factors relevant to intervention efficacy will be informative.

Finally, the review authors’ interpretations of empirical study findings may have influenced our conclusions. While some authors provide cautious statements pertaining to intervention efficacy, others were more confident in their interpretations—even when reviews had substantial study overlap. For instance, there was a 73% overlap in the controlled studies included in Currier and colleagues^1^ and Rosner and colleagues.^39^ The latter review, however, formed more positive conclusions than the former. Future umbrella reviews may consider using meta-analytic strategies to summarize their findings to decrease subjective interpretation of results.

## CONCLUSION

This umbrella review reveals a rather concerning picture. The problem is not that bereavement interventions are shown to be ineffective, but rather that study quality is often so low that conclusions about efficacy cannot be drawn. Methodological shortcomings of the empirical studies are consistently criticized by reviewers. More specifically, the lack of RCTs and follow-up assessments means that reliable conclusions about intervention effectiveness cannot be drawn. Concerns also extend to how reviewers analyze and report their findings. In particular, reviews often fall short in investigating and discussing important quality criteria, such as ROB.

Additionally, a knowledge gap remains concerning when bereavement interventions are effective. Though there has been some examination of relevant moderators, we still have much to learn about what types of treatment are more or less effective, for whom treatment works, and in what situations these variables are relevant. To gather this knowledge, empirical studies should, for example, reduce heterogeneity in terms of participant and intervention characteristics by isolating important variables, such as type of loss, to determine treatment effects for these particular characteristics. This will allow authors of meta-analyses to further explore moderation effects, leading to knowledge about when, and for whom, an intervention works best.

Similarly, our understanding of the mechanisms that explain intervention efficacy remains limited. Notably, none of the reviews adequately focus on mechanisms of treatment efficacy. Understanding these mechanisms is imperative to providing high-quality care to bereaved individuals. Currier and colleagues^1^ made a plea for inspecting cognitive mechanisms, noting that “theory would be advanced by using ‘dismantling designs’ . . . that could assess the critical mechanisms within intervention programs.” Such dismantling designs will allow for us to compare efficacy of important treatment components both separately and in combination, deepening insight into why an intervention is effective or ineffective. Furthermore, to facilitate understanding of which components to target, it is imperative that interventions are guided by theory. Theory-driven interventions will result in a more precise explanation of treatment effects, which in turn will help refine and develop theoretical concepts,^68^ enabling overall improved understanding of how to best support bereaved individuals.

While the results of this umbrella review offer some indications of bereavement intervention efficacy, extensive work is needed to provide clear guidance on implementing these interventions in practice. This review’s results underscore the need for further empirical studies, systematic review articles, and meta-analyses to execute well-designed, high-quality research to arrive at reliable conclusions concerning intervention efficacy for bereaved individuals.
